# Isolation of Intact and Functional Melanosomes from the Retinal Pigment Epithelium

**DOI:** 10.1371/journal.pone.0160352

**Published:** 2016-08-23

**Authors:** Laura Pelkonen, Mika Reinisalo, Emmanuelle Morin-Picardat, Heidi Kidron, Arto Urtti

**Affiliations:** 1 School of Pharmacy, University of Eastern Finland, Kuopio, Finland; 2 Centre for Drug Research, Division of Pharmaceutical Biosciences, University of Helsinki, Helsinki, Finland; Eye Hospital, Charité, GERMANY

## Abstract

Melanosomes of retinal pigment epithelium (RPE) have many vision supporting functions. Melanosome research would benefit from a method to isolate pure and characterized melanosomes. Sucrose gradient centrifugation is the most commonly used method for isolation of RPE melanosomes, but the isolated products are insufficiently characterized and their quality is unclear. Here we introduce a new gentle method for fractionation of porcine RPE that produces intact functional melanosomes with minimal cross-contamination from other cell organelles. The characterization of isolated organelles was conducted with several methods confirming the purity of the isolated melanosomal fraction (transmission electron microscopy, immunoblotting) and presence of the melanosomal membrane (fluorescence staining of melanosomal membrane, zeta potential measurement). We demonstrate that our isolation method produces RPE melanosomes with the ability to generate free phosphate (P_i_) from ATP thereby proving that many membrane proteins remain functional after isolation. The isolated porcine RPE melanosomes represented V-type H^+^ATPase activity that was demonstrated with bafilomycin A1, a specific V-ATPase inhibitor. We anticipate that the isolation method described here can easily be optimized for the isolation of stage IV melanosomes from other pigmented cell types and tissues.

## Introduction

Melanosomes are specialized lysosome-related organelles that contain melanin pigment [1]. Melanin can be further distinguished into brown/black eumelanin and yellow/red pheomelanin, but most natural melanins are a mixture of these two polymers [2]. Melanosomes are found in several organs including the skin, brain, inner ear and in several tissues of the eye, such as the heavily pigmented retinal pigment epithelium (RPE) located in the posterior part of the eye [3]. The RPE consists of a polarized monolayer of cuboidal cells between the neural retina and choroid, and it has many important vision supporting functions. The RPE acts as a part of the blood-retinal-barrier (BRB) maintaining ideal conditions for the neural retina: the RPE provides nutrients to the photoreceptors, takes care of phagocytosis of shed photoreceptor outer segments (POS) and participates in the retinoid cycle that is indispensable for vision [4]. RPE melanosomes, which are composed mainly of eumelanin, have an important role in the absorption of scattered light [2,3]. Additionally, RPE melanosomes protect the neural retina from reactive oxygen species (ROS) by detoxifying free radicals [5] and participate in iron homeostasis [6]. Which other functions the RPE melanosomes may have is currently uncertain.

Melanosome maturation is divided into four stages (I-IV) that can be identified with electron microscopy (EM) [7]. The early stages (I-II), often referred as pre-melanosomes, lack pigment but can be characterized by intraluminal protein fibers that begin to form at stage I and are completed at stage II [8,9]. At stage III, these fibers are thickened by deposited melanin synthesized by tyrosinase, the key enzyme in melanin synthesis. At stage IV, melanin covers all the intraluminal structures and melanosomes can be characterized as highly pigmented ellipsoidal or round organelles surrounded by a membrane bilayer. At mature stage IV tyrosinase activity is not found anymore in the melanosomes [10]. In the RPE, melanosome maturation takes place during fetal development and in early childhood, and therefore, adult RPE contains only mature (stage IV) melanosomes [11].

To enable detailed characterization of melanosomal function, advanced melanosome isolation techniques are required. Isolated RPE melanosomes have been used widely in biological studies [6,12–14], and also in the pharmaceutical field to study melanin binding of drugs [15]. Nevertheless, the term “melanin granule” has been used incoherently to describe either the isolated melanin or isolated organelles, melanosomes, and in many cases, the distinction whether the isolated product represents melanin or melanosomes is unclear. Several nomenclatures were introduced in the 1960’s [16] including a suggestion to describe the most mature form of the granules with no measurable tyrosinase activity as melanin granule [7]. However, today, the term “melanosome” is mainly used with numerical description (I-IV) of different developmental stages of the organelle.

Melanosome isolation methods vary to some extent depending on the tissue of origin [17]. Different methods for distinct developmental stages can be applied for melanosomes from the same origin since their density differs due the varying amounts of pigment [7,10,18]. Sucrose density gradient centrifugation is the most commonly used method for RPE melanosome isolation [17,19] and the method has been used since the 1960’s when it was first applied for melanosome isolation from B16 mouse melanoma cells [7,10]. OptiPrep^®^ gradient centrifugation is also described in the literature for the isolation of RPE melanosomes [20]. In many cases, purified RPE melanosomes are studied with EM to show the integrity of the melanosomal membrane. In addition, other physical methods have been applied in RPE melanosome surface characterization, such as atomic force microscopy [19,21] and scanning electron microscopy [19]. However, sometimes the purified product has not been characterized at all [15,22–24] and therefore, the quality of the organelle after isolation is unclear.

Overall, the isolated melanosome studies are a fragmented field. Many recent studies on isolated RPE melanosomes refer to old purification methods where only physical characterization of the melanosomes was conducted. It is unclear, whether such methods produce purified and functional organelles. Therefore, new isolation method associated with proper characterization of the purified organelles is needed. If isolated organelles are used to mimic the conditions in the cells or tissue, it is crucial that they maintain biological functions, such as active membrane proteins, after isolation. The functionality of isolated RPE melanosomes has been dismissed in the previous studies. Here, we introduce a straightforward isolation method for intact RPE melanosomes with high purity and preserved biological functions.

## Materials and Methods

### Melanosome isolation

The detailed isolation method for porcine RPE melanosomes is described in Supplementary Information (S1 Text). Briefly, porcine eyes were obtained from a local slaughterhouse and they were kept in phosphate buffered saline (PBS) on ice during the transportation. Extraocular material was removed and the anterior part of the eye was removed with a scalpel. The vitreous and neural retina were removed with tweezers and PBS was added into the eyecup. After 5 min incubation at room temperature, the RPE was gently removed with a small paintbrush and collected. Cells were pelleted with 6238 g centrifugation for 5 min and stored at -20°C until further processing.

**Fig 1:**
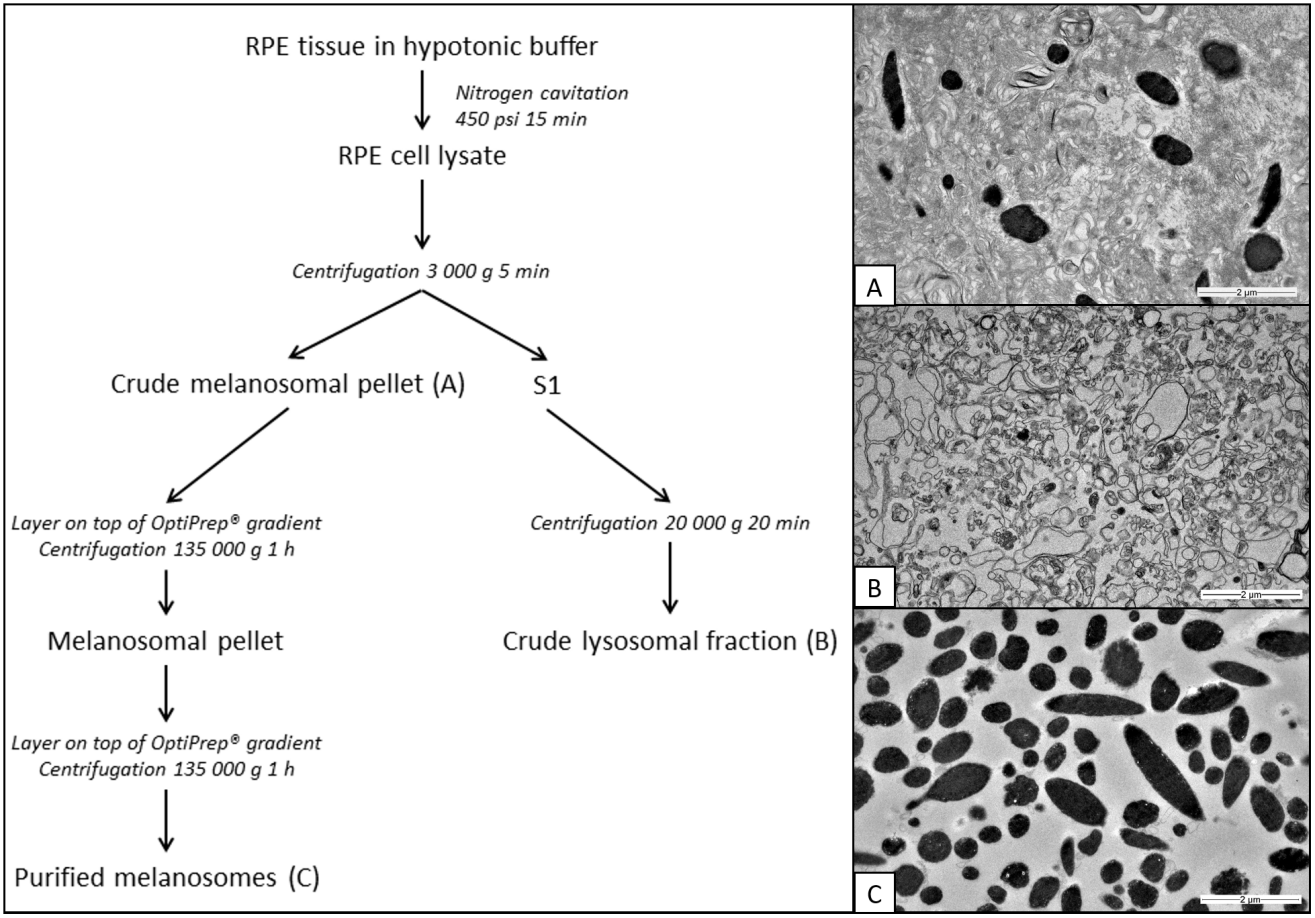
RPE fractionation method and EM images of isolated RPE fractions. A) In addition to melanosomes, the crude melanosomal fraction contains a large quantity of other membranous structures. B) In the crude lysosomal fraction, only few melanosomes were observed among non-pigmented vesicles. C) The purified melanosomal fraction contains mainly round or ellipsoidal melanosomes. Scale bar 2 μm, 6000x magnification.

Melanosomes were isolated using OptiPrep^®^ (Axis Shield, Oslo, Norway) density gradient centrifugation (Fig 1). The RPE tissue was thawed on ice and suspended into hypotonic buffer (10 mM Tris-HCl, 10 mM NaCl, 1.5 mM MgCl_2_) containing inhibitors (PMSF and protease inhibitor cocktail, Sigma-Aldrich, St. Louis, MO, USA). Cells were disrupted with nitrogen cavitation (Parr 4639, Parr Instrument Co., Moline, IL, USA) at 450 psi with 15 min equilibration time. Whole cell lysate was centrifuged at 3000 g for 5 min and the supernatant (crude lysosomal fraction) was removed and pelleted at 20 000 g for 20 min. Crude melanosomal pellet was re-suspended into 50 mM Tris-HCl 150 mM KCl buffer (pH 7.40) and layered on top of discontinuous OptiPrep^®^ gradient. Gradient tubes were centrifuged at 135 000 g for 1 h at + 4°C (Sorvall^™^ WX Ultra Centrifuge, TH-641 Swinging Bucket Rotor, Thermo Fisher Scientific Inc., Waltham, MA USA). The melanosomal pellet was recovered and re-suspended into 50 mM Tris-HCl 150 mM KCl buffer (pH 7.40) and re-centrifuged in another OptiPrep^®^ gradient. After the second gradient centrifugation, melanosomes were re-suspended into MES buffer (25 mM MES, 5 mM NaCl, 115 mM KCl, 1.3 mM MgSO_4_, pH 7.40) and residues of OptiPrep^®^ were removed by two 10 000 g centrifugations. The total protein concentration of the purified melanosomal fraction was measured with the Bradford method (Bio-Rad Protein reagent, Bio-Rad, Hercules, CA, USA).

### Electron microscopy

For the electron microscopic examination, the melanosomal and lysosomal fractions were firstly fixed with 2.0% paraformaldehyde and 2.5% glutaraldehyde (Electron Microscopy Sciences) prepared in 0.1 M cacodylate buffer for 1 h at room temperature. After pre-fixation, the samples were post-fixed with 1% osmium tetroxide in 0.1 M cacodylate buffer for 1.5 h in room temperature and dehydrated through ascending series of ethanol: 70%, 90%, 94% and absolute ethanol, followed by propylene oxide. The samples were then infiltrated in a mixture (1:1) of propylene oxide and epoxy resin for 2 h at RT, followed by pure epoxy overnight. Thereafter, specimens were polymerized for 24 h in 37°C and followed by 48 h at 60°C. Thin sections were cut using Ultracut UC7 ultramicrotome (Leica Microsystems, Wien, Austria) and stained routinely with uranyl acetate and lead citrate. Samples were examined and imaged with JEM-2100F transmission electron microscope operated at 200 kV (Jeol Ltd, Tokyo, Japan).

### ATPase assay

The ATPase assay was conducted with a commercially available ATPase assay Kit (#601–0120, Innova Biosciences Ltd, Babraham, Cambridge, UK) with minor modifications. Briefly, isolated melanosomes were incubated at + 37°C for 30 min with or without 1.6 μM bafilomycin A1 (Sigma-Aldrich) in MES buffer. Then, an equal volume of ATP-containing buffer (1 mM ATP, Innova Biosciences) was added and melanosomes were incubated at room temperature for 30 min. After incubation, melanosomes were removed by centrifugation (20 000 g 5 min) and the supernatant was recovered. Color reagent and stabilizer were added to the supernatant. After 30 min, free phosphate (P_i_) concentrations were measured with a spectrophotometer at A595 nm.

### Western blot

Isolated melanosomal fractions were introduced to 1% Triton-X-100 buffer containing inhibitors (PMSF and protease inhibitor cocktail, Sigma-Aldrich) and disrupted with sonication. Twenty μg of protein was loaded on each well of SDS-PAGE gel (4–20% Precise Tris-Glycine Gels, Thermo Fisher Scientific Inc. Waltham, MA, USA). Proteins were transferred onto PVDF membrane (Bio-Rad) with semi-dry electroblotting (Trans-Blot^®^ SD Semi-Dry Transfer Cell, Bio-Rad). Primary antibody incubations were conducted at +4°C overnight (mouse-anti HSP60 1:1000, #ADI-SPA-806-F, Enzo Life Sciences Inc., Farmingdale, New York, USA; Anti-Na^+^/K^+^ATPase α1, 1:1000, 05–369, Merck Millipore, Merck KGaA, Darmstadt, Germany; Atp6v0a1 antibody, 1:500, GTX44653, GeneTex Inc., Irvine, CA, USA, Rab27a antibody 1:500, 0023, SICGEN–Research and Development in Biotechnology Ltd, Cantanhede, Portugal). Secondary antibody incubations (goat anti-mouse, sc-2005 and goat anti-rabbit sc-2030, 1:10 000, Santa Cruz Biotechnology, Santa Cruz, CA, USA) took place at room temperature for 45 min. Protein-antibody-complexes were detected with chemiluminescence reaction (Image Quant RT ECL, GE Healthcare, Little Chalfont, UK) using Amersham^™^ ECL^™^ Prime Western Blotting Detection Reagent (GE Healthcare).

### Zeta potential and size measurement

Zeta potential and size of the isolated melanosomes were measured in Tris-MES buffer (50 mM Tris, 12.5 mM MES, 2.5 mM MgCl_2_, 2.5 mM NaCl, 57.5 mM KCl, 0.65 mM MgSO_4_, pH 7.40) with Nanosizer ZS instrument (Malvern Instruments Ltd, Malvern, UK) in folded capillary cells (#DTS1070, Malvern Instruments). Two melanosome concentrations (0.1 μg/μl and 0.01 μg/μl) were used.

### Fluorescence staining of melanosomal membranes

Staining of the melanosomal membranes was carried out with lipophilic carbocyanine dye, Vybrant^™^DiO (V-22886, Thermo Fisher Scientific) according to manufacturer´s instruction. Shortly, 5 μg of melanosomes and 1 μg of synthetic melanin (#M8631, Sigma-Aldrich) were stained with 5 μM DiO at + 37°C for 15 min. After staining, melanosomes were washed twice with 1xPBS. After washing, melanosomes were suspended with Mowiol^®^4–88 mounting media (#81381, Sigma-Aldrich) on a microscope slide and imaged at Ex484 nm/Em501 nm by using fluorescence microscope (Axio Imager with ApoTome.2, Carl Zeiss, Oberkochen, Germany) with Carl Zeiss ZEN imaging software.

## Results

### RPE fractionation enables isolation of highly purified melanosomes with minimal contamination of other organelles

**Fig 2:**
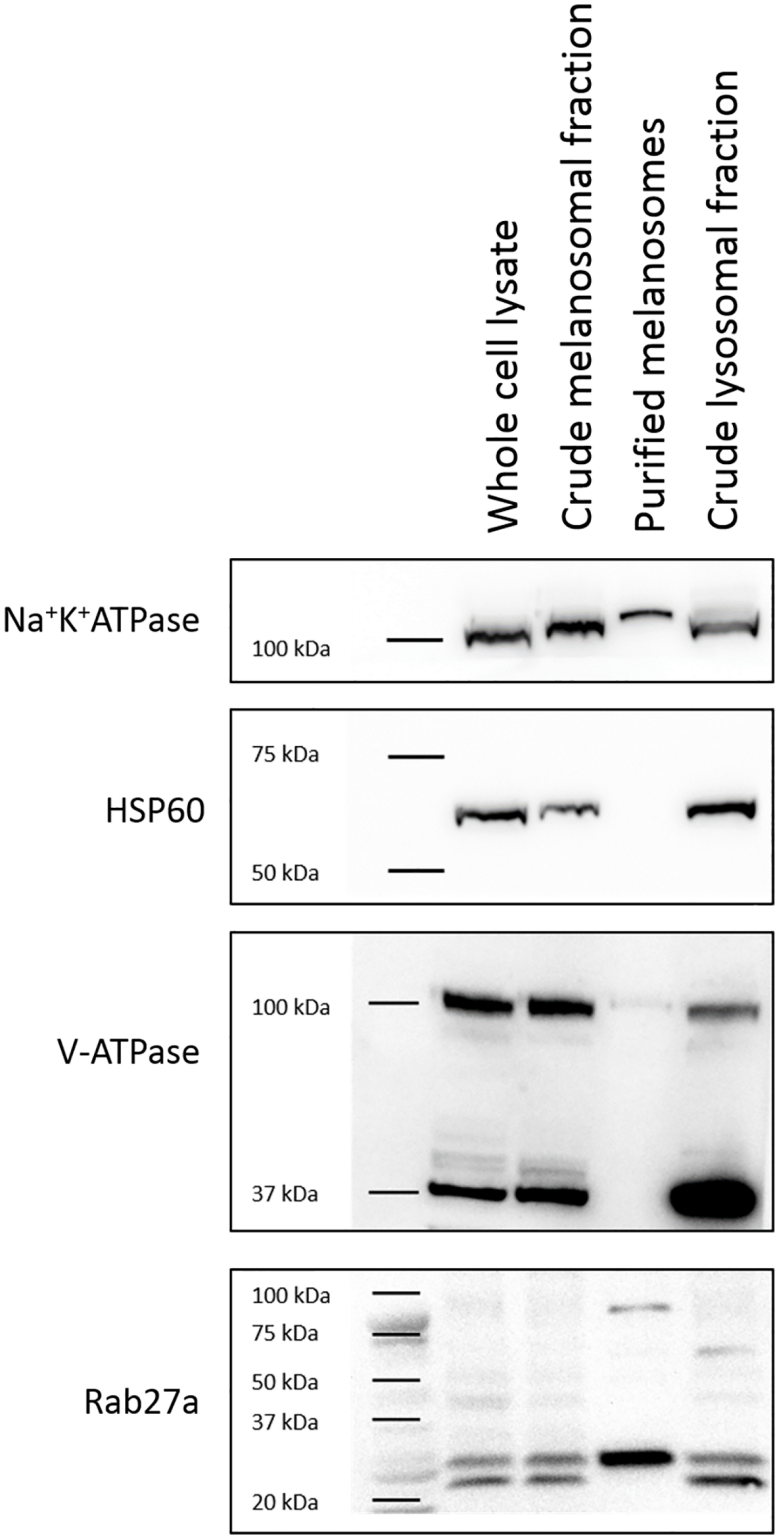
Na^+^K^+^ATPase (112 kDa), HSP60 (60 kDa), V-ATPase (96 kDa) and Rab27a (27 kDa) expression in the porcine RPE melanosomal fractions and in the porcine RPE crude lysosomal fraction. The mitochondrial marker HSP60 is absent in the purified melanosomal fraction whereas the V-ATPase as well as the Na^**+**^K^**+**^ATPase are expressed in all fractions. Rab27a is enriched in the purified melanosomes.

In general, isolation of pigmented RPE melanosomes was easily confirmed visually by eye. The purity of the isolated melanosomes was also verified by using transmission electron microscopy (TEM) (Fig 1, for lower magnification, see S1 Fig) and cell organelle-specific markers (Fig 2).

TEM imaging proved that the purified melanosomal fraction represents exclusively melanosomes free of other cellular organelles such as nuclei, mitochondria and lysosomes (Fig 1C). In the crude melanosomal fraction, a large quantity of both melanosomes and undefined membranous structures were present (Fig 1A) whereas in the crude lysosomal fraction the presence of melanosomes was low displaying mainly membranous structures and non-pigmented vesicles (Fig 1B).

Purity of the fractions was also verified using immunoblot analysis of the mitochondria-specific marker HSP60 as well as transmembrane ATPase markers Na^+^K^+^ATPase and V-ATPase (Fig 2). In addition, Rab27a, a melanosomal membrane marker, was used. HSP60 was present in all fractions except in the purified melanosomal fraction indicating that this fraction was free of mitochondrial contamination. However, Na^+^K^+^ATPase and V-ATPase were found in all fractions, also, at low levels, in purified melanosomal fraction. (Fig 2). Importantly, Rab27a expression was highest in the purified melanosomal fraction.

### Purified melanosomes display intact lipid membrane with ATPase activity

**Fig 3:**
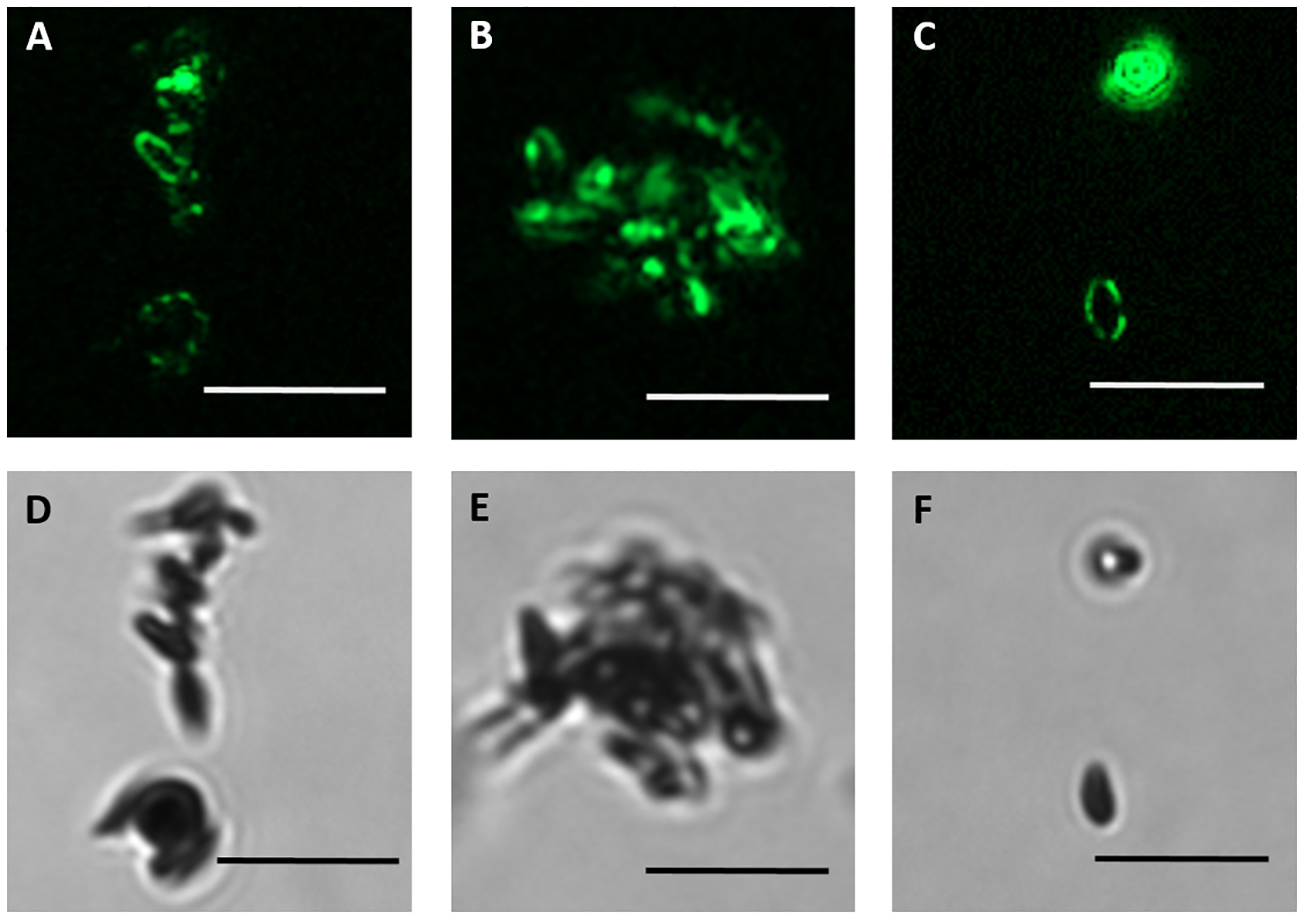
Purified melanosomes display intact membrane structure. Staining of isolated porcine RPE melanosomes with Vybrant^™^DiO (A-C), a fluorescent membrane dye (green) showed that melanosomal membrane remains intact after isolation process. (D-E) represent brightfield images. Scale bar 5 μm.

Fluorescence signal from lipophilic carbocyanine DiO dye demonstrates that isolated melanosomes are surrounded by membrane (Fig 3, for a lower magnification, see S2 Fig). Fluorescence signal was not detected in synthetic melanin (S3 Fig).

**Fig 4:**
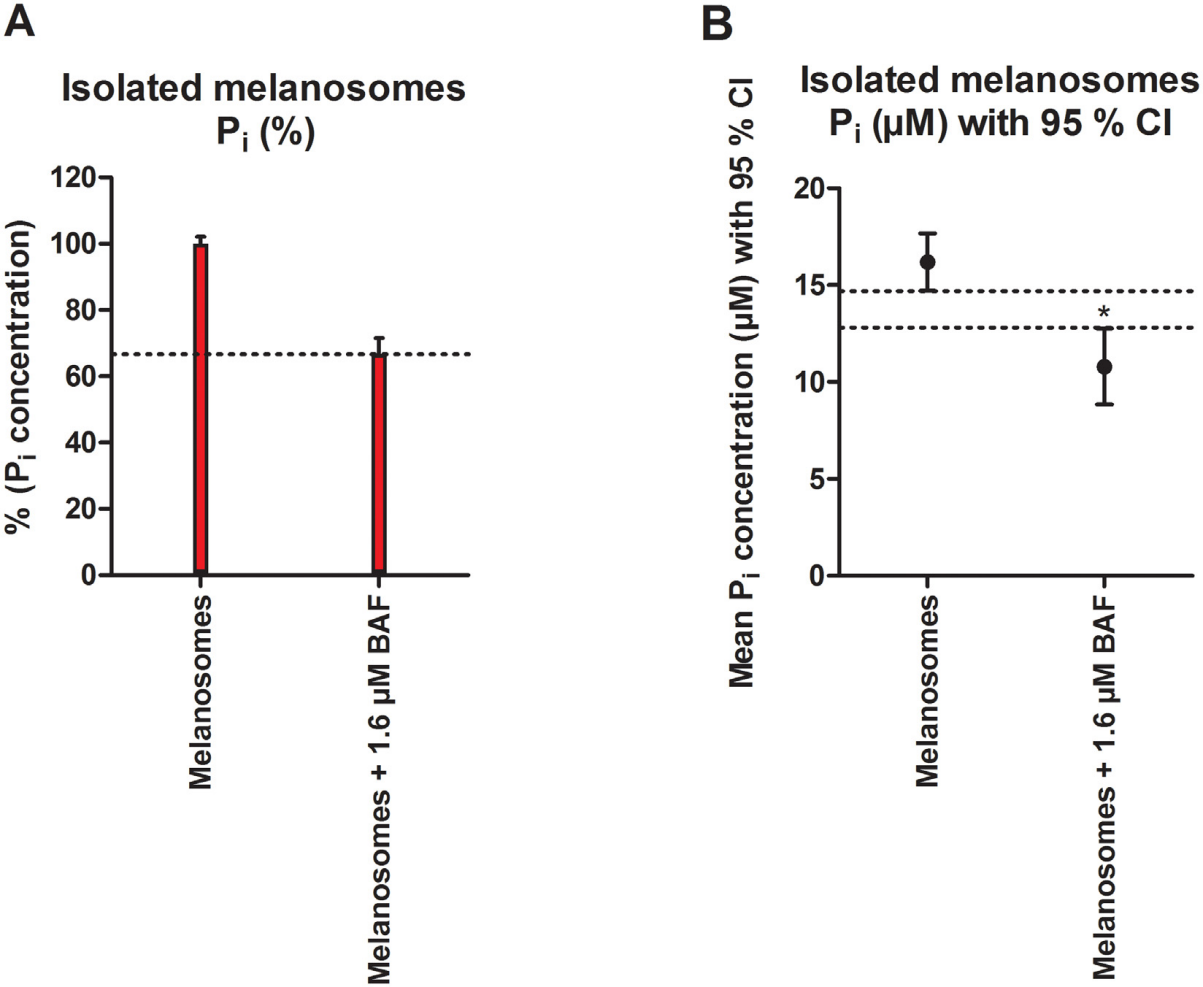
Melanosomes retain their biological activity after isolation. (A) In ATP hydrolysis assay, purified melanosomes were incubated with ATP and concentration of generated free phosphate (P_i_) was measured. Assay was carried out with or without V-ATPase inhibitor bafilomycin A1 (BAF). Values are shown as mean +/- S.E.M (n = 3). (B) Statistically significant difference in P_i_ formation with BAF is shown as 95% Confidence Interval (indicated by dashed lines, *P < 0.001, unpaired t-test).

The ATPase assay revealed that isolated melanosomes can generate free phosphate (P_i_) from ATP proving that biological activity is preserved in the isolation process (Fig 4). Compared to non-treated melanosomes, the reduction in the amount of P_i_ formed with specific V-type H^+^ATPase inhibitor bafilomycin A1 (1.6 μM) was 33.3% (Fig 4A) indicating that V-type H^+^ATPase remains functional after isolation.

Zeta potential values could not be detected from isolated melanosomes, indicating that the strong negative charge of melanin is masked by the melanosomal membrane (S4 Fig).

**Fig 5:**
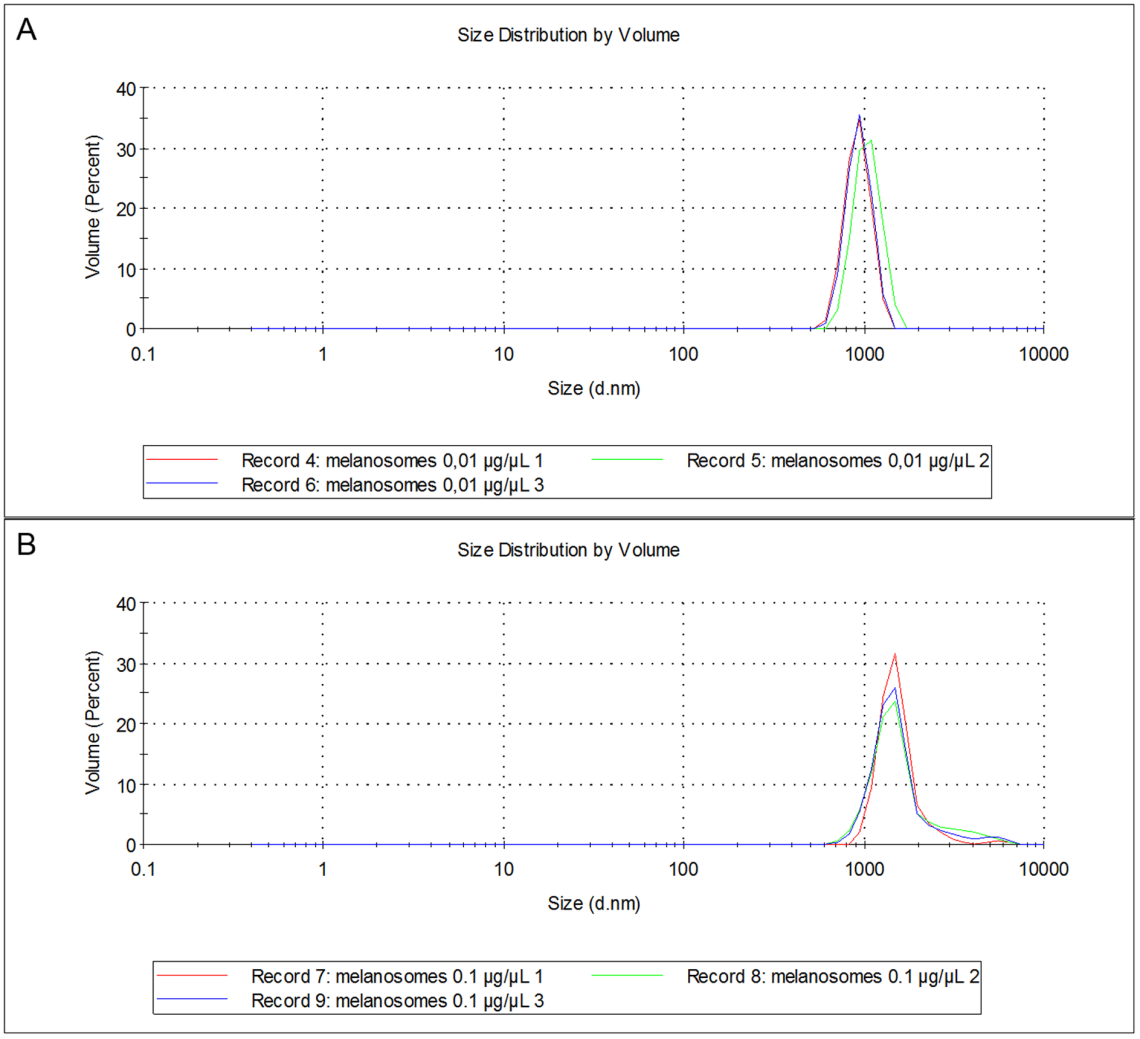
Size analysis of isolated melanosomes. (A) At lower melanosomal concentration 0.01 μg/μl, one population with an average diameter of 934.5 nm was observed whereas at higher melanosomal concentration (B) 0.1 μg/μl, two populations with average diameters 1533 and 5577 nm were observed.

Size analysis at lower melanosomal concentration (0.01 μg/μl, Fig 5A) identified one population with an average diameter of 935 nm and at higher concentration (0.1 μg/μl, Fig 5B), the main population represented an average diameter of 1533, and also a minor population with an average diameter of 5577 nm was observed. The shape of some isolated organelles was found to be round or ellipsoidal in EM images (Fig 2C), which could explain identifying two apparent populations depending on the relative position of the melanosome to the light source. Melanosomal aggregation at higher melanosomal concentration (0.1 μg/μl, Fig 5B) may also explain the observation of two organelle populations.

## Discussion

Our aim was to isolate an intact, functional and pure melanosomal fraction from porcine RPE cells. A new, gentle isolation method that utilizes nitrogen cavitation as a disruption method together with multilayer OptipPrep^®^ gradient centrifugation was developed to ensure the quality of the isolated organelles. This was confirmed by characterizing structural as well as functional features of melanosomes such as intact lipid membrane and preserved activity of membrane protein. The combination of hypotonic buffer and nitrogen cavitation provides a gentle method for cell disruption. In the procedure, friction is not generated and therefore, the organelles and proteins do not undergo heat damage during disruption [25]. Additionally, nitrogen cavitation allows treatment of each cell only once increasing the probability that intracellular organelles remain intact in the disruption procedure. It should be noted that nitrogen cavitation has previously been used in the isolation of intact human RPE lysosomes [26].

OptiPrep^®^ has been used previously to isolate melanosomes from porcine RPE [20,27]. In these studies, melanosomal fraction was layered on top of 50% OptiPrep^®^ after cell debris and light mitochondrial fraction were removed from melanosomal fraction with differential centrifugation. However, in our study, low-speed centrifugation was not applicable to separate cell debris from melanosomal fraction due to the sedimentation of melanosomes. Therefore, a multilayer OptiPrep^®^ gradient was used to separate melanosomes, other organelles and cell debris from crude melanosomal fraction. Several layers ensured effective purification. Azarian et al. reported that the nuclei were most common contaminants in the melanosomal fraction [20]. Therefore, in our gradient, layers of 30% and 35% of OptiPrep^®^ were included, since in nuclei isolation, nuclear fraction is collected between these layers [28].

Melanosomes in general are described as closely-related organelles to lysosomes [1] and both organelles express the proton-transporting V-ATPase [20]. In our study, the main concern was that lysosomes would be present in the melanosomal fraction since they could interfere with the functionality studies due to their V-type H^+^ATPase activity. Since commonly used lysosomal markers cathepsin D [20,29] and Lamp-1 [29] are also found in melanosomes, immunoblotting with the markers is not applicable in distinguishing lysosomes from melanosomes. However, lysosomes are considered to remain in the supernatant containing light mitochondrial fraction after 3000 g centrifugation [30] whereas the 3000 g pellet represents the crude melanosomal fraction in our protocol. After this step, in lysosome isolation protocols, crude lysosomal fraction (or light mitochondrial fraction) is pelleted at 15 000–20 000 g and then further processed for example with OptiPrep^®^ density gradient media. However, layers of more dilute OptiPrep^®^ (8–27%) are used, and according to Sigma-Aldrich’s lysosome isolation kit (#LYSISO1), enriched lysosomal fraction is collected from 16–19% OptiPrep^®^. Based on these differences in the lysosome isolation, the lysosomes should not be a contaminant in the purified melanosomal fraction. This was verified with TEM (Fig 1C).

The size of isolated melanosomes (935–5577 nm, Fig 5) differs slightly from the size of isolated porcine RPE-choroid melanin (0.1 μg/μL: about 800 nm) studied in our group previously with dynamic light scattering (DLS) (data not shown). Pitkänen et al. reported that isolated melanin from porcine RPE-choroid had a diameter of 0.2 to 10 μm [31]. However, aggregation due to the higher melanin concentration (2 μg/μL) together with different analysis method (laser diffraction vs. DLS) might explain the difference. Also in our study, it is possible that melanosomal aggregation occurred and thus, the diameters measured with higher melanosomal concentrations may be overestimated. Previously, in isolated adult bovine RPE melanosomes, the long axis was found to be 0.6–2.3 μm and the short axis was approximately 0.6 μm [13]. Detailed size analysis of porcine RPE melanosomes has not been described in the literature, but our findings show that melanosomes and melanin differ in size.

Zeta potential can be used to assess the nature of the particle surface charge (positive/negative), and it is more widely used in the characterization of nanoparticles [32]. However, since melanin is negatively charged due to the carboxylic groups on its structure [33], the zeta potential investigations have been used to characterize melanin [34] and its interactions with for example proteins [35,36]. Interestingly, zeta potential of isolated porcine RPE-choroid melanin is associated with ambient pH, displaying stronger negative charge at neutral pH 7 (−20.3 mV) compared to more acidic pH 5 (−17.1 mV) [34]. Our study showed that in the isolated melanosomal fraction, the strong negative charge of melanin is not detectable (S4 Fig), and therefore, the isolated product is likely to consist of intact organelles where the strong negative charge of melanin is concealed by the melanosomal membrane. Based on EM images, the integrity of the melanosomal membrane is difficult to evaluate (Fig 2C), whereas the fluorescent membrane dye shows its presence clearly (Fig 3C). The lack of fluorescent membrane signal in synthetic melanin (S3 Fig) indicates, that the membrane caused the staining in the purified melanosomes. Additionally, immunoblotting showed that Rab27a, a membrane protein localized on the melanosomal surface [37], was enriched in the purified melanosomal fraction (Fig 2). This confirms the presence of the melanosomal membrane in purified melanosomes. The fact that membrane proteins are functional after isolation (Fig 4) also indicates that melanosomal membrane remains intact during isolation.

V-ATPase has an important vision supporting role in maintenance of central functions of RPE such as POS phagocytosis and autophagy where it has mainly been connected with its function in RPE lysosomes [38]. Additionally, it was found to have a role in RPE pigmentation in zebrafish [39]. V-ATPase activity has been shown in isolated B16 murine melanoma melanosomes with the usage of specific inhibitors [40]. However, no studies describing the functionality of this or other proteins in isolated RPE melanosomes have been conducted previously. V-type H^+^ATPase has previously been detected from isolated RPE melanosomes with LC-MS/MS among other ATP-binding cassette transporters such as Na^+^K^+^ATPase [20]. We also detected the expression of both of these proteins in purified melanosomes by immunoblotting (Fig 2). V-ATPase regulates the intraorganelle pH, and therefore its functionality is crucial for the quality of isolated organelles [41]. Consequently, a closer investigation regarding the function of this protein was carried out by using ATPase activity assay in combination with bafilomycin A1, a well-known V-type H^+^ATPase specific inhibitor [42]. Since other proteins with ATPase activity may also be present in isolated porcine RPE melanosomes (e.g. Na^+^K^+^ATPase, Fig 2), P_i_ formation in the presence of ATP cannot be abolished with bafilomycin A1 completely.

In conclusion, we introduced a new isolation method for porcine RPE melanosomes, which is gentle, relatively easy and quick, and results in a highly purified melanosomal fraction with intact melanosomal membrane and functional membrane proteins. The isolation method described here can easily be applied for stage IV melanosome isolation from other cell types, and the method can be further modified to produce stage IV melanosomes from tissue. Functionality and integrity of purified melanosomal fraction was evaluated with ATPase assay, EM, zeta potential measurement and fluorescent membrane probes. These methods could all be used to characterize isolated melanosomes from different origin as well. We illustrate a set of characterization methods that could be used routinely to confirm the quality of isolated organelles when a new isolation method is applied. Zeta potential measurements together with ATPase assay are a quick and easy way to ensure that the isolated product consists of functional organelles rather than melanin.

## Supporting Information

S1 Fig Electron microscopy image of the purified melanosomal fraction. The purified melanosomal fraction is enriched with round and ellipsoidal melanosomes and is free from other organelles. Scale bar 2 μm, 3000x magnification.
(TIF)

S2 Fig Vybrant^™^DiO staining of the purified melanosomes shows intactness of the melanosomal membrane. (A) represents brightfield image of isolated porcine RPE melanosomes. A fluorescent membrane dye (Vybrant^™^ DiO, green) showed that melanosomal membrane remains intact after isolation process (B). Scale bar 10 μm.
(TIF)

S3 Fig Synthetic melanin does not give fluorescence signal with the fluorescent membrane dye (green, Vybrant^™^DiO). (A) represents brightfield image showing the location of melanin aggregates. With AF488 channel, background fluorescence was observed (B). (C) Represents both channels showing that fluorescence is not co-localizated with synthetic melanin. Scale bar 20 μm.
(TIF)

S4 Fig Phase plots of purified melanosomal fractions with A) 0.01 μg/μL and B) 0.1 μg/μL concentrations. No zeta potential was observed in the purified melanosomes.
(TIF)

S1 Text Melanosome isolation method. (DOCX)
