# Supplementary figures and images for: Isolation of Intact and Functional Melanosomes from the Retinal Pigment Epithelium
Source: PLoS One. 2016 Aug 23;11(8):e0160352. doi: 10.1371/journal.pone.0160352 (PMC4994940; doi:10.1371/journal.pone.0160352)

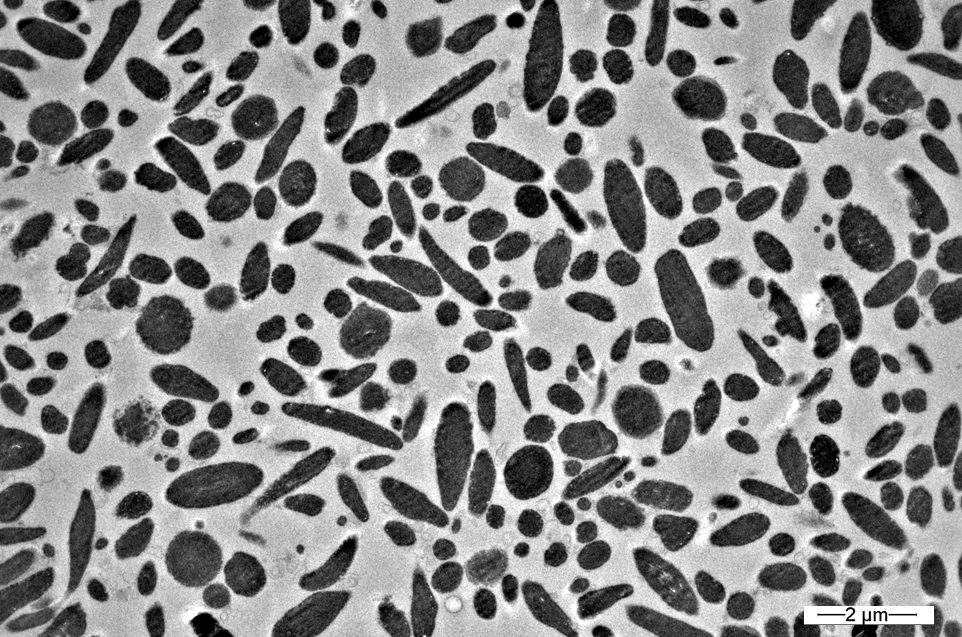

Supplement: S1 Fig — The purified melanosomal fraction is enriched with round and ellipsoidal melanosomes and is free from other organelles. Scale bar 2 μm, 3000x magnification. (TIF) [file pone.0160352.s001.tif]

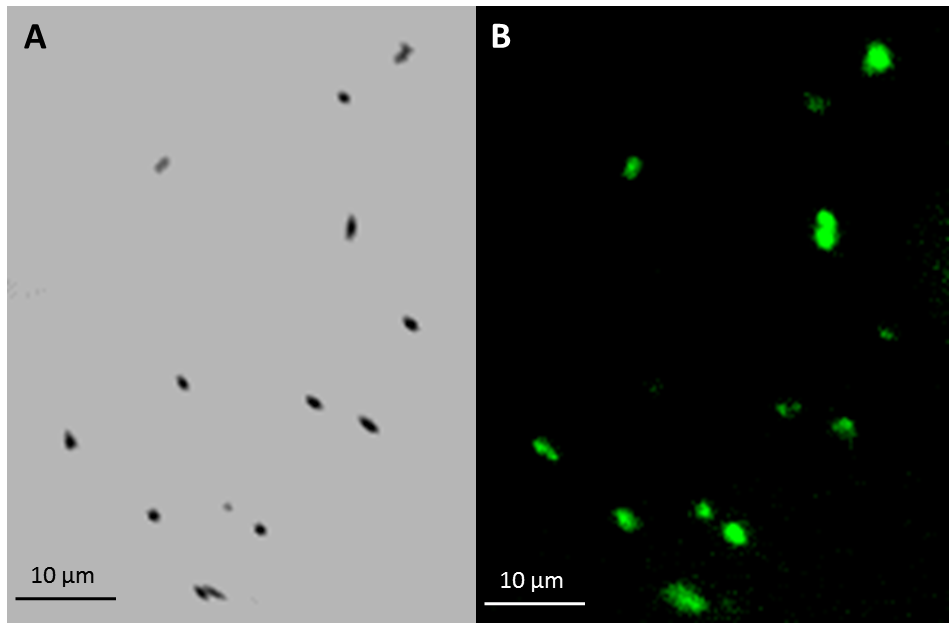

Supplement: S2 Fig — (A) represents brightfield image of isolated porcine RPE melanosomes. A fluorescent membrane dye (Vybrant™ DiO, green) showed that melanosomal membrane remains intact after isolation process (B). Scale bar 10 μm. (TIF) [file pone.0160352.s002.tif]

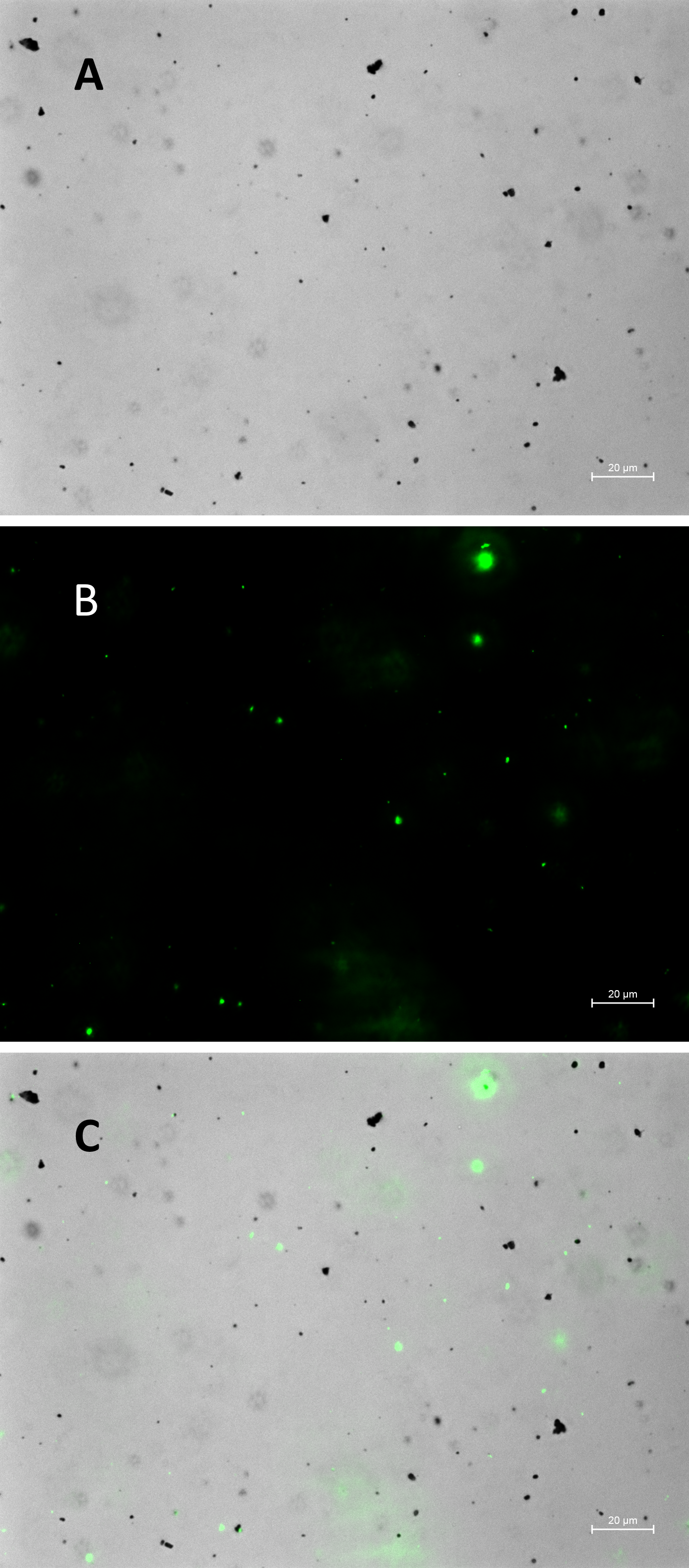

Supplement: S3 Fig — (A) represents brightfield image showing the location of melanin aggregates. With AF488 channel, background fluorescence was observed (B). (C) Represents both channels showing that fluorescence is not co-localizated with synthetic melanin. Scale bar 20 μm. (TIF) [file pone.0160352.s003.tif]

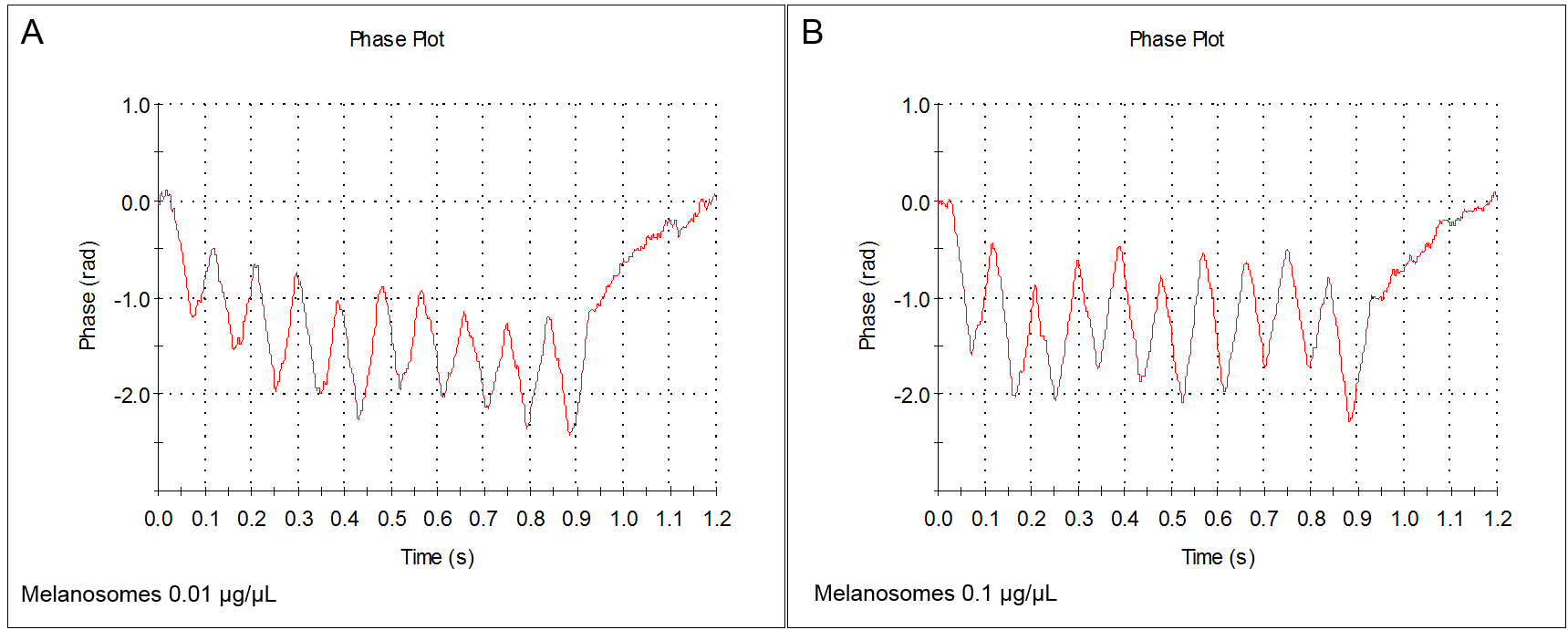

Supplement: S4 Fig — No zeta potential was observed in the purified melanosomes. (TIF) [file pone.0160352.s004.tif]
