# Supplementary material for: Isolation of Intact and Functional Melanosomes from the Retinal Pigment Epithelium
Source: PLoS One. 2016 Aug 23;11(8):e0160352. doi: 10.1371/journal.pone.0160352 (PMC4994940; doi:10.1371/journal.pone.0160352)
Supplement: S1 Text — (DOCX) [file pone.0160352.s005.docx]

**S1 Text. RPE melanosome isolation method**

**Reagents**

**PBS:** 10xDPBS (#14200-067, Gibco BRL, Grand Island NY, USA) diluted into 1xDPBS with MilliQ H_2_O

**Hypotonic buffer:** 10 mM Tris-HCl, 10 mM NaCl, 1.5 mM MgCl_2_

**125 mM PMSF solution:** Dilute 21.8 mg PMSF (#P7626, Sigma-Aldrich, St. Louis, MO, USA) into 1 ml of 2-propanol

**Protease inhibitor cocktail:** #P8340, Sigma-Aldrich, St. Louis, MO, USA

**Homogenization medium:** 0.25 M sucrose, 25 mM KCl, 5 mM MgCl_2_, 20 mM Trisine-KOH, pH 7.40

**Tris buffer:** 10 mM Tris-HCl, 150 mM KCl, pH 7.40

**OptiPrep®:** 60 % iodixanol solution (Axis Shield, Oslo, Norway)

Dilute into 50 % OptiPrep Working solution with Homogenization medium

Make dilutions of 35 %; 30 %; 20 % and 15 % by diluting 50 % OptiPrep Working solution with Tris-buffer

**MES buffer:** 25 mM MES, 5 mM NaCl, 115 mM KCl, 1.3 mM MgSO_4_, pH 7.40

**Equipment**

**Scalpels** Model 11, #L503 Lance Paragon Ltd., Sheffield, England, UK

**Scissors**

**Tweezers**

**Small paintbrush**

**Petri dishes**

**18 G needles #**NN-1838R, Terumo Medical Corporation, Somerset, NJ, USA

**2.0 mL syringes** CODAN Medizinische Geräte GmbH & Co KG, Lensahn, Germany

**22 G needles** #NN-2238R, Terumo Medical Corporation, Somerset, NJ, USA

**Nitrogen cavitation device** Parr 4639, Parr Instrument Co., Moline, IL, USA

**Table centrifuge** Hettich Mikro 22R, Hettich Instruments, LP., Tuttlingen, Germany

**Ultracentrifuge tubes** Sorwall^TM^ Polyallomer tubes 13.2 ml, Thermo Fisher Scientific Inc., Waltham, MA USA

**Ultracentrifuge** Sorvall^TM^ WX Ultra Centrifuge, Thermo Fisher Scientific Inc., Waltham, MA USA

**Swing-Out Rotor** TH-641 Swinging Bucket Rotor, Thermo Fisher Scientific Inc., Waltham, MA USA

**Porcine RPE isolation**

Porcine eyes should be removed immediately after sacrificing the animal. Eyes need to be kept on cold PBS (on ice) during transportation and at all stages of isolation (unless otherwise mentioned).

Remove the extraocular material using scissors and tweezers. Dip the eye into 70 % ethanol and place the eye into fresh PBS solution.

Transfer the eye onto a large petri dish. Make a cut approximately 2 mm behind the limbus with sharp tipped scalpel. Remove the anterior part of the eye with scissors, and pour out the vitreous from the eyecup. Remove the neural retina with round-tipped tweezers and place the eyecup onto 12 well plate. Add 1 mL of cold PBS into the eyecup and let it stand in room temperature for 5 min.

Detach the RPE with small paintbrush and collect cells into 50 mL tube. Rinse the eyecup twice with PBS in order to collect all cells.

Pellet the cells at 6238 g for 5 min at + 4 °C. Remove the supernatant and store RPE cells as pellets at – 20 °C until further processing.

**Melanosome isolation**

Pre-cool the vessel chamber of Parr Instrument nitrogen cavitation device in the fridge or on ice for 30 min.

Thaw RPE cells on ice and keep the sample on ice during all the isolation steps.

Add inhibitors (125 mM PMSF solution and protease inhibitor cocktail) into hypotonic buffer: 10 µL inhibitor/1 mL buffer.

Suspense RPE cells into hypotonic buffer: 1 mL buffer/RPE tissue from 5 eyes.

Make holes into the caps of 1.5 mL Eppendorf tubes with 18 G needle.

Transfer the homogenate into 1.5 ml Eppendorfs with holes in the caps (1 mL of homogenate/tube).

Place the RPE tubes into the vessel of nitrogen cavitation device. Place the chamber on ice and adjust the pressure to 450 psi and equilibrate for 15 min. Disrupt the cells by releasing the nitrogen after 15 min.

Transfer the cell lysate into fresh 1.5 mL Eppendorf tubes

Take a whole cell lysate sample if necessaire.

Centrifugate the cell lysate at 3000 g for 5 min at + 4 °C.

Collect the supernatant (S1) into fresh 1.5 mL Eppendorf tubes.*

Resuspense the crude melanosomal pellet into 1 mL of Tris-buffer.

Take a crude melanosomal fraction sample if necessaire.

Prepare the discontinous OptiPrep® gradient using 13.2 ml Polyallomer tubes (Sorwall^TM^):

´ Pipet 1.5 mL of 50 % OptiPrep® solution to the bottom of the tube.

Layer carefully following OptiPrep® layers on top of the solution using 2.0 mL syringe and 22 G needle:

1.5 mL of 35 % OptiPrep®

1.0 mL of 30 % OptiPrep®

1.5 mL of 20 % OptiPrep®

2.0 mL of 15 % OptiPrep®

Layer the crude melanosome fraction carefully on top of 15 % OptiPrep® with pipette (crude melanosomal fraction from five RPEs into one gradient tube).

Centrifugate the gradient tubes at 135 000 g for 1 h at + 4 °C (Sorvall^TM^ WX Ultra Centrifuge, TH-641 Swinging Bucket Rotor, Thermo Fisher Scientific Inc., Waltham, MA USA).

Prepare identical OptiPrep® gradient tubes as above.

After centrifugation, discard all upper layers and resuspense the melanosomal pellet into 1 mL of Tris-buffer.

Layer the melanosomal pellet onto 15 % OptiPrep® and re-centrifugate the gradient tubes at 135 000 g for 1 h at + 4 °C.

After centrifugation, discard all the upper layers and re-suspense the purified melanosomal pellet into 1 mL of MES-buffer and transfer the melanosome suspension into fresh 1.5 mL Eppendorf tube. Fill up the tube with MES-buffer.

Remove the remaining OptiPrep® by two consecutive centrifugations at 10 000 g for 5 min at +4 °C.

Store the melanosomal pellet until further use at – 20 °C.

*Centrifugate S1 at 20 000 g for 20 min at + 4°C (Hettich Mikro 22 R).

Discard the supernatant and store the pellet i.e. crude lysosomal fraction at – 20 °C until further processing.

For use, melanosomes were thawed on ice and re-suspended to appropriate buffer and the total protein concentration of purified melanosomal fraction was measured with Bradford method (Bio-Rad Protein reagent, Bio-Rad, Hercules, CA, USA).
